# Study on Mechanical Properties of Alkali-Activated Coal Gasification Slag Concrete

**DOI:** 10.3390/ma18143240

**Published:** 2025-07-09

**Authors:** Rongjian Shen, Xiaojun Li, Shen Li

**Affiliations:** 1 Shanxi Anjian Investment and Construction Co., Ltd., Xi’an 710003, China; 18391867275@163.com; 2 College of Safety science and Engineering, Xian University of Science and Technology, Xi’an 710054, China; 15991609639@163.com

**Keywords:** coal gasification slag, alkali-activated, mechanical properties, testing method

## Abstract

Coal gasification slag (CGS) is a solid byproduct generated during coal gasification. Stacking and land-filling of CGS wastes substantial land resources and has significant environmental risks. In this paper, based on the Ca/Si and Si/Al ratios of the raw materials, the mix design of alkali-activated CGS concrete was optimized using a pure center-of-gravity design method. The compressive and flexural strengths of geopolymer concrete with varying mix proportions were measured to investigate the effects of sodium silicate modulus, material content, and dry density on its mechanical properties. Specimens of different sizes were prepared to analyze the influence of testing methods on the compressive, flexural, and tensile properties. The results indicate that the mechanical properties of geopolymer concrete are significantly influenced by the raw material composition and the modulus of the activator. With increasing curing age, both compressive and flexural strengths exhibit varying degrees of improvement. The stress-strain behavior of alkali-activated CGS concrete aligns closely with that of ordinary concrete. A comparative analysis of 100 mm length and 20 mm length cubic specimens revealed a compressive strength size conversion coefficient of approximately 0.456, while the flexural specimen exhibited a coefficient of 0.599. For tensile strength evaluation, both the Brazilian splitting method and the double punch test method yielded consistent and reliable results, demonstrating their suitability for assessing CGS-based concrete.

## 1. Introduction

China is the largest producer and consumer of the coal in the world [1]. The advancement of coal conversion technologies toward higher efficiency, lower energy consumption, and minimized environmental impact has become an inevitable trend in energy development [2]. Researchers have focused on finding new low-carbon cementitious materials that can replace Portland cement, and promoting the green transformation of coal [3]. Among them, coal gasification is the main method for clean utilization of coal, but a large amount of CGS is produced in the process of gasification. CGS is a primary solid byproduct of coal chemical processes and poses significant environmental and health risks when improperly managed [4]. The research on comprehensive development and utilization of CGS has great ecological and economic significance, aiming to avoid environmental pollution, and promote green development.

Regarding the utilization of CGS, scholars have conducted extensive research on its applications in construction and highway engineering fields, and have also explored its potential uses in the ceramic industry [5]. Based on the potential resource value of CGS, scholars have conducted much research on the physical properties and chemical components of CGS. The mineral composition of CGS is similar to that of FA, and it is rich in Si, Al and Ca, which has potential pozzolanic activity, resulting in the potential to replace cement in the preparation of composite cementitious materials [6]. Furthermore, the coarse slag contains more aluminate, silicate and other mineral phases. In the process of hydration with cement or hardening with lime, hydrated calcium silicate and calcium aluminate gel with dense structures are formed, which improves the strength and durability of cement paste [7]. Li et al. studied the characteristics of hydration products of CGS and cement and showed that a high content of residual carbon in fine slag inhibits the hydration reaction between CGS and cement. The residual carbon in the coarse slag is lower, and the active oxides such as SiO_2_ promote the hydration reaction, produce more gel, and improve the compressive strength of the cement mortar [8]. Li et al. [9] prepared thermally conductive UHPC with CGS. Lian et al. [10] investigated the reaction mechanism of CGS in relation to cement; in contrast to CGFS, the presence of abundant active mineral phases in CGCS promoted the cementitious reaction and improved the strength of the mortar. Jiao et al. [11] found that substituting quartz sand and river sand with CGS (possessing a higher crushing value) in UHPC mixtures led to increased compressive strength under standard curing. The maximum strength occurred when 75% of the conventional fine aggregates were replaced. Yoshitaka [12] added CGS as an aggregate to concrete and tested the aggregate performance and mechanical properties of the concrete, reporting that the 28 d compressive strength of CGS concrete and natural sand concrete showed no difference. ZHAO et al. [13] found that ceramsite prepared by cement, quartz and CGS according to a certain ratio can be used as concrete aggregate and admixture. Yang et al. [14] discussed the feasibility of using gasification slag as auxiliary cementitious material (SCM) and systematically studied the working performance, mechanical properties and microstructure characteristics of cement paste after adding gasification slag. The results show that the compressive strength of cement paste with gasification slag is about 3.7–9.3% lower than that of ordinary Portland cement (PC), but it can still meet the design requirements of 42.5 composite Portland cement. Fu et al. [15] demonstrated that when the CGS content is below 10%, it can effectively function as both nucleation sites and pozzolanic material in cement paste. Compared to ordinary Portland cement (PC) without CGS addition, the G1P specimen exhibited 7.1%, 6.9%, and 5.4% increases in compressive strength at 1 d, 7 d, and 28 d, respectively. The unreacted gasification slag primarily exists in aggregated form. Luo et al. [16] systematically investigated the effects of coal gasification coarse slag (CGA) and fine slag (CGS) on cement paste hydration and microstructure development, determining their strength activity indices to be 100.9% and 82.7%, respectively. Current research on utilizing CGS as supplementary cementitious materials (SCMs) remains limited, and large-scale application as SCMs proves difficult without prior carbon-ash separation treatment. According to Wei et al. [17], CGS functions effectively as a raw material in nonburnt brick production, with elevated dosages yielding products compliant with the MU20 standard [18]. Separately, Zhang et al. [19] determined that CGS pozzolanic mineral composition boosts mortar strength in alkaline settings, qualifying it as a sustainable alternative to conventional fine aggregates and partial cement replacement in roadway applications.

In summary, scholars have carried out research on different engineering applications of CGS, but as yet, there is no unified understanding for the rational and effective use of CGS. In this study, using differently sized specimens, the compressive strength, flexural strength and tensile strength of concrete were studied. The influence of different mix ratios and specimen sizes on the mechanical properties of concrete was studied, and the evaluation system of the mechanical properties of concrete was further improved, which has reference value for the utilization of CGS.

## 2. Materials and Methods

### 2.1. Raw Materials

#### 2.1.1. Cementitious Materials

CGS: CGS is a solid waste byproduct of coal gasification methanol production. The CGS used in this study was sourced from a chemical company in Shanxi Province, China. The sieve analysis results and physical properties of the material are presented in Table 1 and Table 2 [10], respectively.

According to the preliminary screening statistics, the particles with a particle size of less than 2.36 mm in the CGS account for about 73%, particles of 2.36–4.75 mm account for about 21%, and the rest are particles with a particle size of more than 4.75 mm 20.

**Supplementary cementitious materials**: FA, SF, and limestone flour (LF) were used as supplementary cementitious materials to produce geopolymer concrete. The chemical composition of the binders is listed in Table 3 [10].

#### 2.1.2. Alkali Activator

In this experiment, sodium silicate is used as an activator to configure the cementitious materials; a sodium silicate solution produced by a chemical plant in Wuxi was used as the activator. The chemical composition and basic properties of the sodium silicate are shown in Table 4.

Chemical analysis grade, pure granular sodium hydroxide with a purity of 99% was used to adjust the modulus of the sodium silicate solution. The sodium silicate modulus (Na_2_O·nSiO_2_) represents the molar ratio of SiO_2_ to Na_2_O in the sodium silicate solution, where n is the modulus of sodium silicate. By adding NaOH particles to the sodium silicate solution, the sodium silicate modulus is adjusted to that required for each mix ratio. The principle is shown in Formula (1).

(1) $$Na_{2}SiO_{3}+2NaOH=Na_{2}SiO_{3}+Na_{2}O+H_{2}O$$

The amount of NaOH added in 100 g sodium silicate solution was determined according to Formula (2).

(2) $$n=\frac{26.1/60}{7.96/62+x/80}=\frac{0.435}{0.128+x/80}$$

where 26.1 is the content of SiO_2_ in the solution, and 60 is its molecular weight; 7.96 is the content of Na_2_O in the solution, and 62 is its molecular weight; and 80 is the molecular weight of 2 mol NaOH.

### 2.2. Specimen Preparation

The preparation procedure was conducted as follows: Step 1: The sodium silicate solution was prepared one day in advance according to the required modulus and cooled for subsequent use. Step 2: The binders were mixed at low speed for 2 min. Step 3: After thorough mixing, the aggregates were added and stirred for 1 min. Step 4: The sodium silicate solution and water were slowly poured into the mixer, followed by slow stirring for 7 min and then rapid stirring for 1 min. Step 5: The mixture was poured into molds and vibrated for 1 min on a vibration table, after which the surface was sealed with plastic film for moisture retention; the test blocks in their molds were placed in a standard curing room maintained at (20 ± 2) °C with 95% relative humidity for 48 h, then demolded and continuously cured until reaching the predetermined age.

### 2.3. Test Methods

#### 2.3.1. Compressive Strength Testing Method

In this experiment, cubic test samples with side lengths of 20 mm and 100 mm were used to test the compressive strength of alkali-activated CGS concrete. After 48 h of pouring, the test block was demolded, and the conventional curing of 7 d, 14 d and 28 d was carried out to test the cube compressive strength of concrete at three ages [20].

(3) $$f_{c}=\frac{F_{c,\max}}{A}$$

where *f_c_* represents the compressive tensile strength of the concrete cube specimens; *F_c,max_* is the failure load of the specimen; and A is the bearing area of the specimen.

#### 2.3.2. Flexural Strength Testing Method

The test samples of 20 × 20 × 80 mm and 40 × 40 × 160 mm were used to test the flexural strength. After 48 h of pouring, the test blocks were demolded and cured at room temperature for 7 d, 14 d and 28 d. The flexural strength of concrete at three ages was tested. The flexural strength was calculated according to Formula (4):

(4) $$f_{f}=\frac{1.5PL}{b^{3}}$$

where *f_f_* represents the flexural strength of the concrete specimen; *P* is the failure load of the specimen; L is the distance of two fulcrums; and b is the side length of the prism square section.

#### 2.3.3. Tensile Strength Testing Method

(1) Brazilian splitting method

Tensile properties represent crucial mechanical characteristics of geotechnical materials. The tensile strength of concrete represents its capacity to withstand tensile fracture under tensile stress. In this study, the Brazilian splitting method and double punch test were employed to investigate the tensile strength of alkali-activated CGS concrete with various mix proportions.

The Brazilian splitting [21] method involves placing a cylindrical specimen horizontally at the center of the testing machine’s bearing plate. Two loading baselines are drawn along the axial direction on the specimen’s side, and two spacers are aligned along these baselines. When loaded in compression, the specimen experiences tensile failure along its radial direction, enabling the determination of its tensile strength. The calculation formula is as follows:

(5) $$\sigma_{t}=\frac{2p}{\pi DH}$$

where is the maximum tensile stress at the specimen’s center, MPa; *p* represents the failure load, N; D is the specimen diameter, mm; and H is the specimen height, mm.

The test specimen, with a diameter (D) of 50 mm and height (H) of 25 mm (height-to-diameter ratio of 0.5), complies with the specification requirements for cylindrical specimens (diameter range: 48–54 mm; height-to-diameter ratio: 0.5–1.0).

(2) Double punch testing method

**Figure 1:**
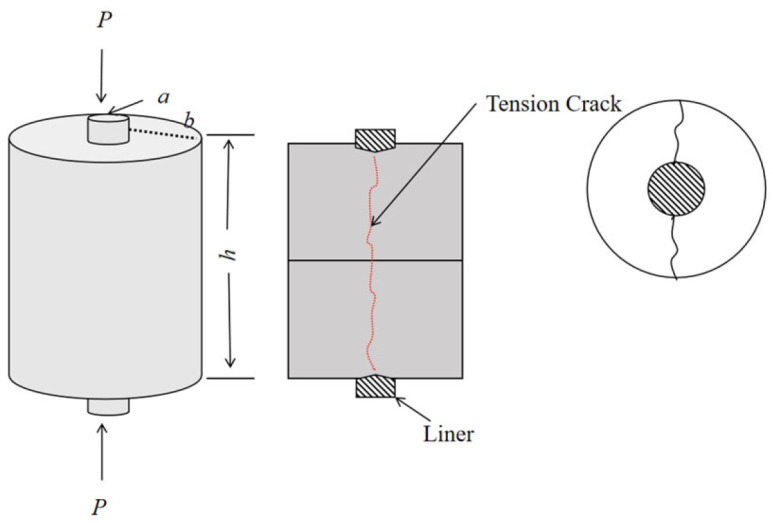
Double punch test layout and failure diagram.

The double punch test [22] can indirectly determine the tensile strength of geotechnical materials. Fang and Chen proposed a new tensile test method based on the radial fracturing mechanism, which is called the double punch test. The layout is shown in Figure 1 [22].

The test method to determine the tensile strength involves vertically positioning the cylindrical specimen between the press’s bearing plates with cylindrical steel pads serving as liners at both ends, then applying an axial load until tensile failure occurs. Based on limit analysis and experimental data, the calculation formula is as follows:

(6) $$\sigma_{t}=\frac{P}{\pi \left(kbh-a^{2}\right)}$$

where σₜ represents the specimen’s tensile strength (MPa), P denotes the ultimate pressure at failure (kN), b is the specimen radius (m), H stands for the specimen height (m), a indicates the liner diameter (m), and k is the material constant (approximately 1.0 according to experimental measurements on concrete, mortar, asphalt cement, and rock).

The cylindrical specimens were designed with a diameter-to-height ratio of 1:1.15 and a short-cylinder liner diameter-to-specimen diameter ratio of 1:4. For this test, the actual dimensions were as follows: specimen radius b = 30 mm, height H = 70 mm, and liner diameter a = 15 mm. These all meet the specified dimensional requirements.

#### 2.3.4. Particle Size Distribution

The particle size distribution of the materials was tested according to the sieve analysis method specified in “Test Methods of Aggregate for Highway Engineering” (JTG 3432-2024) [23], specifically following the T0327-2005 fine aggregate sieve analysis procedure. The testing was conducted using the square-hole sieves prescribed in the standard

#### 2.3.5. SEM Tests

Scanning electron microscopy (SEM, VEGA I-XMU, TESCEN, Prague, Czech Republic) was used to examine the effect of the mix ratio on the mechanical properties of specimens along with the modulus of activator.

### 2.4. Mix Ratio Design

The pure centroid design method is an effective approach for optimizing the composition of concrete materials. In this study, this method was employed to optimize the relative components of the concrete mixture. Based on a review of the relevant literature and preliminary experiments, the principles for configuring CGS concrete using sodium silicate as an alkali activator were established. The process is as follows:

(1) Selection of applicable supplementary cementitious materials: In the process of preparing geopolymer concrete and determining the mix ratio, the selection of raw materials, especially cementitious materials, will have an important impact. Cement, FA, SF, lime, slag and red mud are commonly used in the production of cementitious materials; the main components and alkali-binder ratio will affect the performance of the concrete. Based on the design principle of alkali-activated cementitious material composition, FA, SF and LF were selected as supplementary cementitious materials. FA is a kind of low-calcium volcanic ash material, which can provide Si and Al elements in the reaction to promote the hydration reaction and generate C-A-S-H as the main reaction product. The addition of LF increases the content of CaO in the reaction system, and then increases the contents of Ca/Si in C-S-H, which makes it easier for the hydration and alkali excitation reactions to occur. The silicon element in SF can promote a more complete polymerization reaction, thereby enhancing the strength of geopolymer concrete. Additionally, due to the small particle size of SF, the unreacted particles can fill the voids in the concrete and increase its density. This improvement in density not only further enhances the strength of the concrete but also significantly improves its resistance to permeability.
(2) Selection of appropriate alkali activator: The selection of activators significantly influences both the early-strength development and long-term durability of materials. Current commonly used activators are primarily categorized into three types: alkali activators, salt activators, and acid activators. Research indicates that sodium silicate as an alkali activator offers distinct advantages: silicon-aluminum ratio modulation, and reaction optimization. Sodium silicate provides soluble silicates that effectively increase the Si/Al ratio in cementitious systems, facilitating formation of more stable geopolymer network structures. The active SiO_2_ and Al_2_O_3_ in FA and SF dissolve in alkaline environments and combine with [SiO_4_]^4−^ from sodium silicate, accelerating the formation of three-dimensional N-A-S-H gel that enhances early strength. Pozzolanic reaction enhancement: The alkaline environment (pH ≈ 13) of sodium silicate rapidly activates depolymerization of glassy phases in FA, releasing reactive silicon-aluminum components, while the high specific surface area of SF increases reactive interfaces, enabling synergistic geopolymerization and cement hydration that reduces setting time and improves interfacial transition zone density.
(3) The adequate utilization of CGS: The process of configuring concrete also follows the principle of environmental protection and relevant design indicators. The proportion of CGS in cementitious materials should be between 20% and 50% to maximize the utilization and consumption of CGS.
(4) Determining the mix ratios of Si/Al and Ca/Si: Based on the prior literature, theoretical calculations and analyses indicate that the optimal performance range for cementitious materials is achieved when the Si/Al ratio is maintained in the range of 2.8–3.5, and the optimal range for the Ca/Si ratio is 0.3–0.5 [24]. Building upon this foundation, this study designed a ternary cementitious system primarily composed of CGS, FA, and SF. While the Si/Al ratio of the system met the target range, the Ca/Si ratio consistently remained below the lower design threshold of 0.3 due to insufficient calcium content. To address this limitation and optimize the system’s composition, LF was introduced as a supplementary calcium source. The modified cementitious system components, along with their corresponding Si/Al and Ca/Si ratios, are presented in Table 5. This adjustment successfully elevated the Ca/Si ratio to the desired range of 0.3–0.5, thereby enhancing the system’s performance.

Determination of the basic experimental parameters: According to the literature and based on previous experiments [25], the water-binder ratio was set to 0.35, and the cement-sand ratio was set to 1.0. When the modulus of sodium silicate is 1.0–2.0, the performance of concrete is the better. Therefore, activators with moduli of 1.0, 1.5 and 2.0 were used in this experiment. The mix ratio scheme is shown in Table 6.

## 3. Results and Discussion

The compressive and flexural strengths of the specimens prepared using the 15 mix proportions are presented in Table 7.

### 3.1. Compressive Strength Performance

#### 3.1.1. Effect of Raw Materials on Compressive Strength

Table 7 shows the effective compressive strength of 15 groups of alkali-activated CGS concrete at 7 d, 14 d and 28 d. It can be seen from the table that when the modulus is 1.0, the ratio of CGS and SF has a significant effect on the compressive strength of concrete. When the ratio of CGS is 0.5–0.6 and the SF is 0.1–0.15, the compressive strength of concrete is the smallest, about 18.98–19.62 MPa. Taking the test results of fourth group of mix proportions as an example, the content of CGS slag is 0.56, the proportion of SF is 0.13, and the compressive strength of the concrete is 18.99 MPa. When the modulus of the activator is 1.5, the effect of SF on the compressive strength of concrete is more significant, and with increases in SF content, the compressive strength also increases. When the ratio of CGS, FA and SF is 0.4, 0.35 and 0.25 respectively, the compressive strength of concrete is the largest. When the modulus is 2.0, the effect of SF on the compressive strength of concrete is still significant. However, as the proportion of SF increases, the strength of concrete decreases gradually. When the proportion of SF increases from 0.13 to 0.19 and 0.23, the compressive strength of concrete decreases from 24.61 MPa to 18.27 MPa and 16.06 MPa.

**Figure 2:**
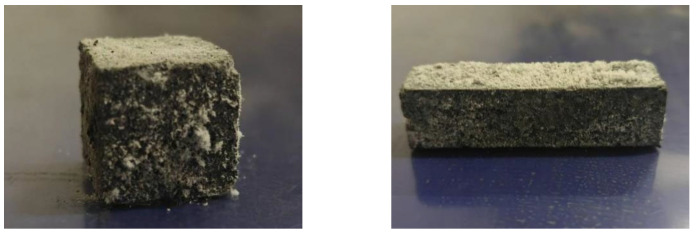
Concrete with efflorescence phenomenon.

As shown in Table 7, the compressive strength of concrete in Groups 1, 3, and 5 increases with decreasing sodium silicate modulus. This trend can be attributed to the higher alkali concentration in the system at lower modulus, which enhances the dissolution of quartz in CGS and FA. As a result, the concentrations of Ca, Si, and Al increase, promoting the formation of N-A-S-H and C-(A)-S-H gels. These gels densify the microstructure, thereby improving the mechanical strength. The addition of NaOH reduces the sodium silicate modulus and modifies its electric double-layer structure, expanding the diffusion layer. This increases the availability of Na⁺ and OH^−^ ions in the system, leading to more thorough activation of SiO_2_ and Al_2_O_3_ in the cementitious materials. Consequently, the polymerization reaction becomes more extensive, contributing to higher compressive strength. Furthermore, elevated alkalinity facilitates the complete excitation of Ca, Si, and Al, increasing the precursor content in the system. This results in a more robust skeletal structure from polymerization, further enhancing the concrete’s strength. However, excessive modulus reduction intensifies the polymerization reaction, causing C-A-S-H gels to encapsulate unreacted particles and form a dense protective film. While this film improves short-term strength, it inhibits further reaction of internal particles and may lead to pronounced efflorescence (Figure 2). This phenomenon explains why some mixtures exhibit reduced compressive strength at very low moduli.

#### 3.1.2. Effect of Curing Age on Compressive Strength

**Figure 3:**
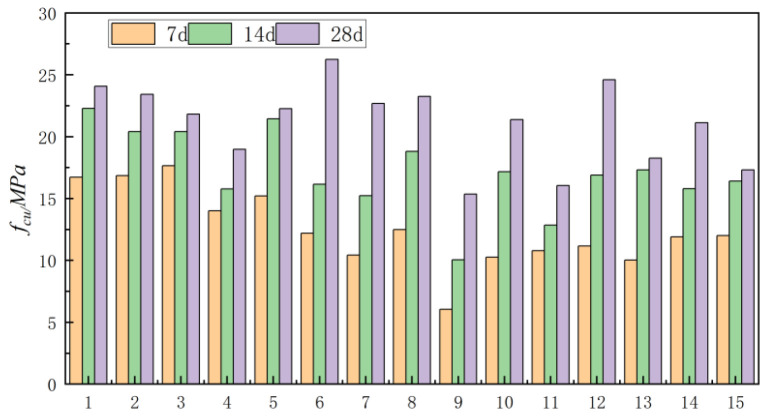
Variation in compressive strength of concrete with curing ages of 7, 14 and 28 days.

Figure 3 shows the variation in compressive strength with curing age for alkali-activated CGS concrete.

The strength development exhibits a distinct two-stage growth pattern: a rapid increase during the early stage (7–14 days), followed by a slower growth rate in the later stage (14–28 days). Group 1 demonstrated a 33% strength increase during the 7–14-day period, approximately 25 percentage points higher than its 14–28-day growth. The most significant difference was observed in Group 13, which showed a 72% strength gain in the first week, 67 percentage points higher than its subsequent growth. In contrast, Groups 4, 11, and 14 exhibited slightly higher strength development during the 14–28-day period, with increases of 8, 6, and 1 percentage points respectively compared to their early-stage growth, indicating relatively stable performance after initial curing.

This behavior can be explained by the reaction mechanisms of alkali-activated materials. In the initial stage, the dissolution of Al and Si oxides from the cementitious material leads to the breaking of Al-O and Si-O bonds in the glassy phase, releasing [AlO₄]⁵^−^ and [SiO₄]^4−^ tetrahedra. These species rapidly polymerize to form geopolymer networks dominated by Si-O-Al and Si-O-Si linkages, contributing to early strength development. As curing continues, the ongoing decomposition of oxides generates increasing amounts of N-A-S-H and C-(A)-S-H gels. The formation of C-(A)-S-H further promotes the dissolution of Si and Al from raw materials, accelerating the geopolymerization process. Due to its more compact structure compared to N-A-S-H, C-(A)-S-H enhances the continued strength gain, though at a reduced rate in later stages [26].

Additionally, the early rapid strength development is facilitated by the substantial exothermic heat released during the dissolution of cementitious materials and sodium silicate, which drives the geopolymerization reaction. However, as the reaction progresses, the heat release diminishes, and in systems with lower modulus, efflorescence occurs while the alkaline environment weakens. These factors collectively result in the observed slower strength growth rate during the later curing period compared to the initial stage.

#### 3.1.3. Size Effect on Compressive Strength

To investigate the size effect on concrete compressive strength, this study prepared 100 × 100 × 100 mm cube specimens using mix the proportions 1–3, 6–8, and 11–13, with three replicates for each mix design. Following standard curing procedures, all specimens were maintained for 28 days before undergoing uniaxial compressive strength testing. This experimental approach enabled systematic evaluation of how specimen dimensions influence measured compressive strength characteristics.

**Figure 4:**
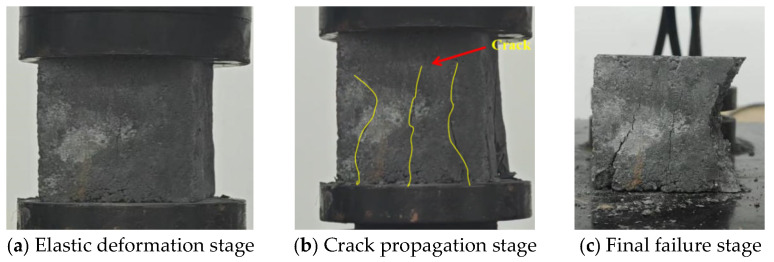
The failure mode of a specimen is three stages.

Figure 4 illustrates the typical failure modes of the specimens. The failure process can be distinctly divided into three characteristic stages: the elastic deformation stage, crack propagation stage, and final failure stage. During the initial elastic stage, the specimens exhibited only elastic deformation under progressively increasing load without visible macroscopic damage. Upon entering the crack development stage, primary penetrating cracks first formed along the principal stress direction, subsequently triggering rapid expansion of multi-directional secondary cracks. The ultimate failure stage manifested as rapid deterioration after peak stress was reached, featuring spalling of the concrete surface layers accompanied by crushing failure in the core region.

The unconfined compressive strength results for the nine mix proportions are presented in Table 8. The data reveal that the 100 mm specimens consistently demonstrate lower compressive strength compared to the 20 mm specimens. This size effect primarily stems from the presence of initial microcracks in aggregates that are inevitably introduced during specimen preparation. Larger specimens contain a greater quantity of aggregates per unit volume, consequently incorporating more inherent microcracks which ultimately reduce the measured compressive strength. The calculation shows that when the modulus of sodium silicate is 1.0, f*_cu_*, 100/f*_cu_*, 20 is about 0.60. When the modulus of sodium silicate is 1.5, the ratios are about 0.31, 0.33 and 0.50, respectively. When the modulus of sodium silicate is 2.0, the ratios are 0.34 and 0.38, indicating that the modulus of sodium silicate will affect the compressive strength of differently sized specimens.

To verify the mechanical properties of CGS concrete, a comparative analysis was conducted with ordinary Portland cement concrete (OPC). The experimental results showed that the compressive strength of CGS geopolymer concrete ranged from 5.61 to 14.83 MPa, while OPC concrete with the same curing age and similar mix proportions typically achieved strengths of 20–40 MPa. Further research will be conducted to optimize the CGS mix ratio to enhance its mechanical performance.

**Figure 5:**
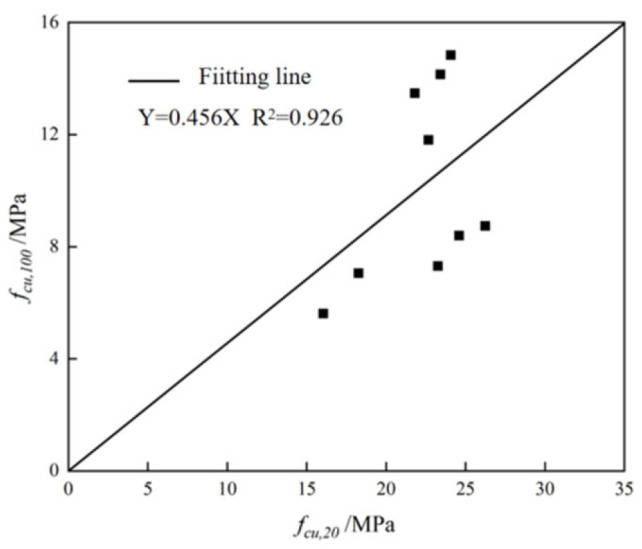
Fitting results of 28-day compressive strength of 20 mm and 100 mm cube specimens.

Figure 5 presents the correlation analysis between compressive strength measurements of 100 mm and 20 mm cube specimens across all nine mix proportions. The experimental data yield a size conversion coefficient of approximately 0.456, indicating a consistent strength reduction relationship between the two specimen dimensions. This empirically derived coefficient quantitatively characterizes the scale effect observed in the alkali-activated concrete system, where larger specimens exhibit proportionally lower compressive strength values compared to their smaller counterparts.

### 3.2. Flexrual Strength Performance

#### 3.2.1. Flexural Strength

Table 7 presents the flexural strength test results under different mix proportions. When the activator modulus is 1.0, the flexural strength of concrete shows distinct trends based on component ratios. With CGS content between 0.35 and 0.50, increasing the SF proportion enhances flexural strength. For instance, comparing Groups 1 and 2 in which the CGS proportions are 0.20 and 0.38 respectively, and the SF proportions are 0.23 and 0.13, the flexural strength increases from 4.42 MPa to 5.95 MPa with higher SF content. Conversely, when CGS content ranges from 0.50 to 0.65, increasing FA content reduces flexural strength, as demonstrated by Groups 4 and 5 in which FA proportions of 0.25 and 0.31 correspond to strength reductions from 5.92 MPa to 3.28 MPa. The minimum flexural strength (3–4 MPa) occurs when CGS is 0.45–0.55, FA 0.3–0.4, and SF 0–0.15. At an activator modulus of 1.5, FA content significantly influences flexural strength when the CGS proportion is 0.35–0.45, with strength decreasing as FA increases. Groups 7 and 8 exemplify this trend, showing a strength reduction from 7.49 MPa to 5.76 MPa as FA decreases from 0.50 to 0.38. Meanwhile, for CGS proportions of 0.40–0.55, SF content becomes the dominant factor, with Groups 8 and 9 demonstrating a strength reduction from 7.49 MPa to 4.67 MPa as SF increases from 0.13 to 0.19. When the modulus reaches 2.0, FA and SF primarily govern flexural strength behavior. For FA proportions of 0.35–0.55, increasing CGS content decreases strength, as shown by Groups 13 and 14 in which strength drops from 9.31 MPa to 6.13 MPa as CGS increases from 0.44 to 0.56. However, with FA proportions of 0.25–0.35, flexural strength improves with increasing SF content.

The test results demonstrate that the flexural strength of concrete is significantly influenced by the modulus of the activator, showing a clear increasing trend with higher modulus values. For instance, the flexural strengths of Groups 4, 9, and 14 were measured at 3.28 MPa, 4.67 MPa, and 6.13 MPa, respectively, exhibiting consistent enhancement as the activator modulus increased. Notably, concrete specimens prepared with an activator modulus of 1.0 consistently exhibited lower flexural strength compared to those with a modulus of 2.0. This behavior can be attributed to several mechanistic factors. When the alkali activator modulus is too low, the sodium silicate solution releases fewer free Si-based geopolymer groups, which inhibits optimal gel formation. Additionally, excessive alkali content leads to the crystallization of NaOH on the surface of geopolymer concrete during hydration, resulting in efflorescence. This surface deposition hinders further hydration reactions, ultimately compromising strength development. Furthermore, as the modulus decreases, the geopolymerization reaction proceeds more completely, forming either a cross-linked network or a layered structure within the matrix. While this enhances reaction completeness, it also increases the brittleness of the resulting geopolymer concrete, which may contribute to the observed reduction in flexural strength at lower moduli.

#### 3.2.2. Effect of Curing Age on Flexural Strength

**Figure 6:**
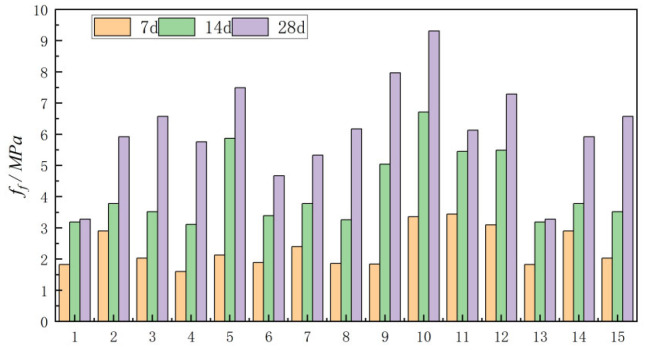
Variation in flexural strength of concrete with curing ages of 7, 14 and 28 days.

Figure 6 illustrates the development of flexural strength of alkali-CGS concrete with curing age. It can be seen in the diagram that the flexural strength growth rate of concrete in the early stage (7–14 d) is faster, while the growth rate in the later stage (14–28 d) is significantly lower than that in the early stage. The strength of the Group 1 concrete increased by 58% during the 7–14 d period, which was about 39 percentage points higher than that during the 14–28 d period. The strength of the Group 12 specimens with 7–14 d of curing increased by 173%, which was about 115 percentage points higher than that of specimens with 14–28 d of curing, and this difference was the largest in Group 15.

#### 3.2.3. Size Effect on Flexural Strength

To investigate the size effect on concrete flexural strength, 40 × 40 × 160 mm prism specimens were prepared using mix proportions 1–3, 6–8, and 11–13, with three replicates for each mix design. All specimens were tested according to standard measurement methods, with flexural strength determined through three-point bending tests.

**Figure 7:**
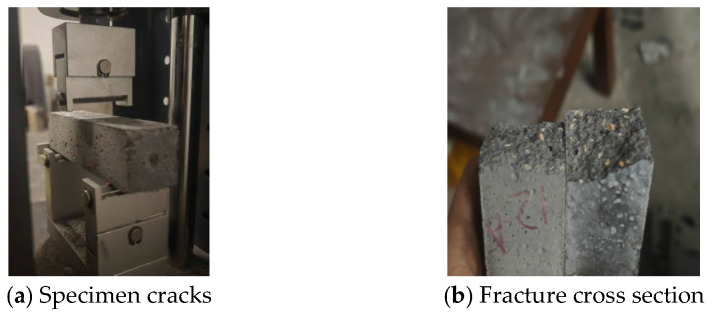
Specimen failure mode.

Figure 7 illustrates the failure mode of the flexural specimens, showing typical brittle fracture characteristics. During loading, no visible cracks appeared in the specimens prior to failure. Upon reaching peak load, the specimens fractured suddenly, demonstrating complete brittle failure behavior.

The results show that the highest flexural strength was observed for sample 1 (5.11 MPa), while sample 3 showed the lowest flexural strength of 3.29 MPa. Based on previous experimental data analysis, the calculated flexural strength ratios between 20 × 20 × 80 mm and 40 × 40 × 160 mm specimens demonstrate clear modulus-dependent characteristics: For specimens with a sodium silicate modulus of 1.0, the flexural strength ratio ranges between 0.84 and 0.89. At a modulus of 1.5, the ratio decreases to approximately 0.60–0.69. With a modulus of 2.0, the ratio further reduces to 0.4.53. These results revealed that the size effect becomes more pronounced with increasing sodium silicate modulus and that the ratio variation follows a consistent downward trend with modulus elevation

**Figure 8:**
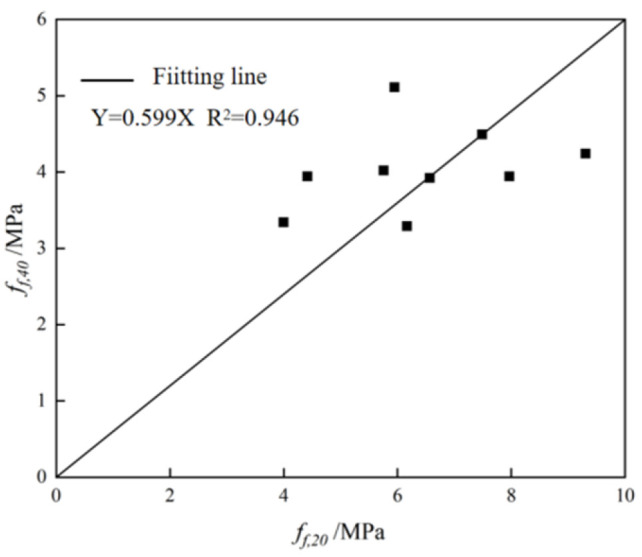
Flexural strength fitting results of 28-day flexural strength for 40 mm × 40 mm × 160 mm and 20 mm × 20 mm × 80 mm specimens.

Figure 8 shows the fitting result of the flexural strength for nine groups of 20 × 20 × 80 mm and 40 × 40 × 160 mm cubes, and the size conversion coefficient is about 0.599.

### 3.3. Tensile Strength Performance

Tensile strength is a crucial mechanical property of geotechnical materials. The tensile strength of concrete refers to its capacity to resist tensile fracture under tensile stress. Based on the fifteen groups of mix proportions determined in the preliminary stage, three moduli were selected, and the three groups of mix proportions were investigated using the Brazilian splitting and double punch test methods to examine the tensile strength of alkali-activated CGS concrete under different mix proportions.

#### 3.3.1. Brazilian Splitting Test

**Figure 9:**
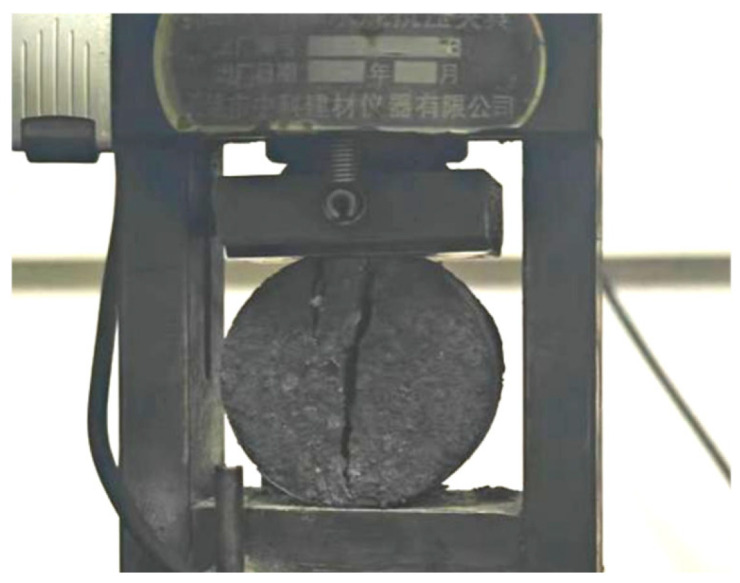
Brazilian splitting specimen failure pattern.

The test specimens were fabricated with dimensions of D = 50 mm and H = 25 mm, meeting the dimensional requirements specified in relevant standards. Figure 9 demonstrates the specimen failure pattern, exhibiting characteristic radial fracture propagation.

The specimens cured to 28 d were placed on the compressive testing machine, and then the radial load was applied to the specimens. When the specimens were damaged, the ultimate pressure was recorded, and the tensile strength of the concrete was calculated according to Formula (5). Three parallel tests were carried out for each mix ratio, and the average value was taken. The Brazilian splitting test was carried out on nine effective specimens, and the measured tensile strength values (MPa) were as follows: 1.80, 1.81, 1.85, 1.72, 1.72, 1.71, 1.64, 1.64, and 1.61. The tensile strength of this group of specimens is in good agreement with the typical variation range of brittle materials.

**Figure 10:**
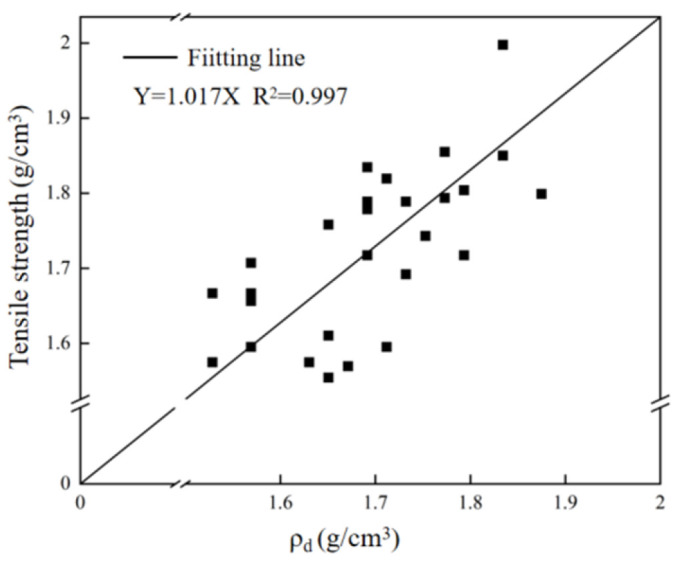
Fitting results of tensile strength and dry density measured by Brazilian splitting method.

Figure 10 shows the linear fitting results of Brazilian splitting tensile strength and dry density for all test specimens. It can be seen from the figure that the fitting result is a straight line passing through the origin, and the dry density is proportional to the tensile strength.

#### 3.3.2. Double Punch Test

**Figure 11:**
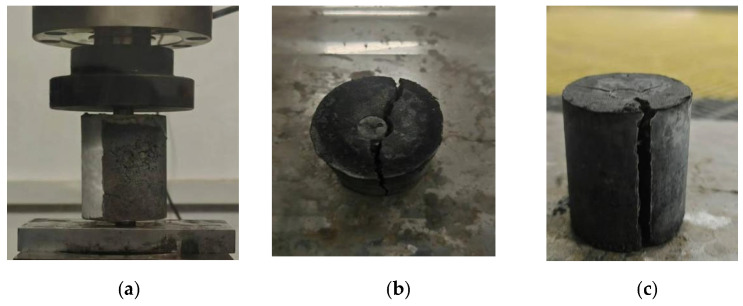
Specimen failure pattern diagrams: (**a**) double punch test diagram, (**b**) axial failure mode of specimen, (**c**) tensile crack.

The double punch test can indirectly measure the tensile strength of geotechnical materials. Fang and Chen proposed this new tensile test method based on the radial fracturing mechanism. The ratio of the diameter to the height of the cylinder sample is 1:1.15, and the ratio of the diameter of the short column liner to the diameter of the specimen is 1:4. The size of the test specimen (b = 30 mm, H = 70 mm, a = 15 mm) meets the required size. The biaxial penetration test diagram and the failure pattern of the specimen are shown in Figure 11.

The specimens with a curing age of 28 days were placed on a compressive testing machine, and then the axial load was applied to the specimens for testing. When the specimens were destroyed, the ultimate pressure was recorded. The tensile strength of the concrete was calculated according to Formula (6). Three parallel tests were carried out for each group of mix ratios, and the average value was taken. The results show that sample 2 had the highest strength with 1.43 MPa, and the lowest flexural strength was observed for sample 13 with 1.20 MPa. The average tensile strength based on the double punch test was 1.33 MPa.

**Figure 12:**
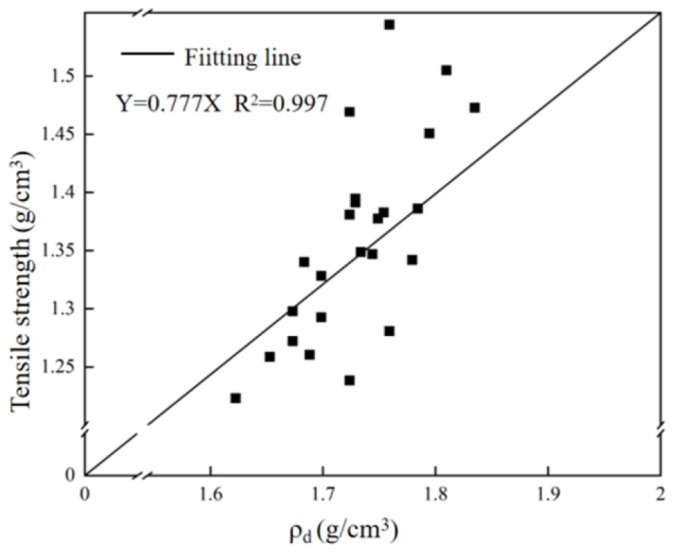
Fitting results of tensile strength and dry density measured by double punch test.

Figure 12 shows the linear fitting results of biaxial penetration tensile strength and dry density of all test specimens. It can be seen from the figure that the dry density is proportional to the tensile strength.

#### 3.3.3. Comparison of the Two Testing Methods

**Figure 13:**
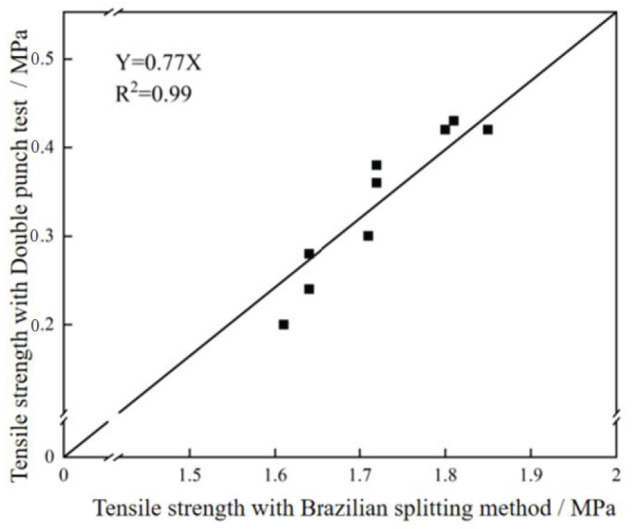
Fitting results of tensile strength measured by Brazilian splitting test and double punch test.

The correlation analysis of the tensile strength measurement results measured by the above two methods was carried out using the analysis software, and the fitting results are shown in Figure 13. It can be seen that the tensile strength measured by the Brazilian splitting and double punch tests has a high degree of correlation, and the tensile strength ratio of the Brazilian splitting and double punch tests is about 0.77. This shows that the results obtained by these two indirect methods for measuring the tensile strength of concrete are basically reliable and can truly reflect the tensile strength characteristics of concrete materials.

#### 3.3.4. Calculation of Shear Strength by Compression-Tension Test

Fang and Hirst first proposed the method of determining shear strength parameters by a compression-tension test. Then, Li and Dong deduced the calculation formula of c (cohesion) and *φ* (internal friction angle) based on the Mohr-Coulomb criterion [27].

(7) $$n=\frac{q_{u}}{\sigma_{t}}$$

(8) $$\sin\varphi =\frac{n-2}{n+2}$$

(9) $$c=\sigma_{t}\cdot \sqrt{\frac{n}{2}}$$

where q*_u_* is the compressive strength, and σ_t_ is the tensile strength determined by the double punch testing method.

The shear strength of concrete refers to its capacity to resist structural failure under shear stress, which is one of the crucial indicators for evaluating the mechanical properties of concrete. According to the theory of material mechanics, the shear strength of unreinforced concrete is typically 10–20% of its compressive strength. From the calculation results presented in Table 9, it can be observed that the data obtained in this investigation generally fall within this theoretical range, thereby preliminarily validating the rationality of the computational approach. However, the actual shear strength of concrete is influenced by numerous factors, including the material composition, curing conditions, and loading patterns. Therefore, to obtain precise shear strength parameters, it is recommended that subsequent research employ direct shear tests or triaxial shear tests to acquire more reliable experimental data.

### 3.4. Microstructure Analysis

In order to investigate the relationship between the microstructure and macroscopic mechanical properties of CGS concrete, microscopic analyses of three groups of CGS concrete with activator moduli of 1.0, 1.5, and 2.0 were carried out. The samples studied were Nos. 1, 6, and 11 in Table 6. The microstructure of concrete was studied by scanning electron microscopy (SEM).

**Figure 14:**
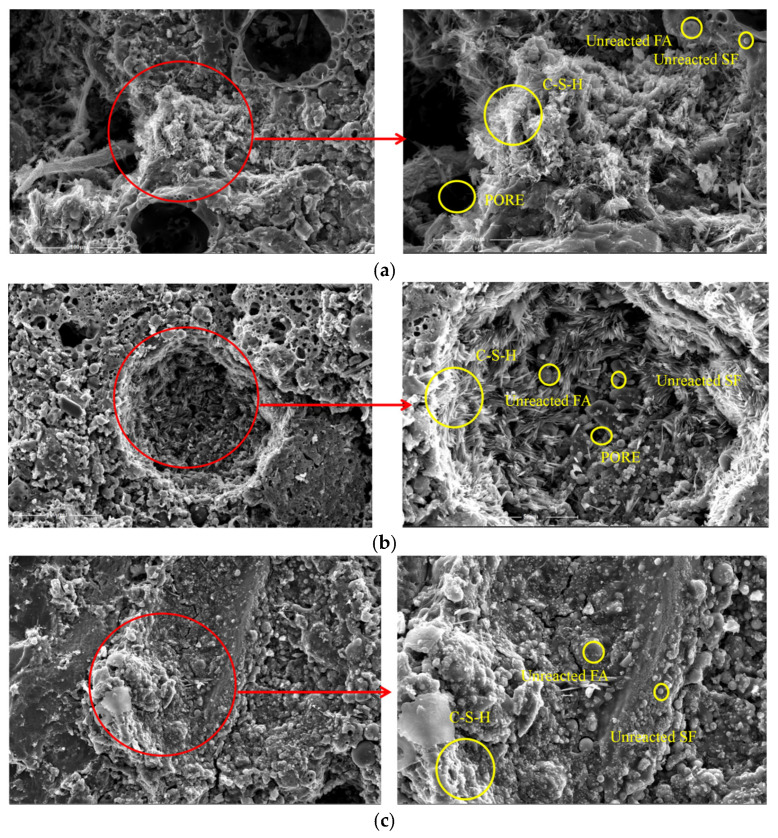
SEM images of samples with different sodium silicate moduli (M): (**a**) M1.0 at 1000× and 2000×; (**b**) M1.5 at 1000× and 2000×; (**c**) M2.0 at 1000× and 2000×.

Figure 14 shows the SEM analysis images of CGS concrete at 1000 and 2000 times. The formation and development of micro-morphology such as interfacial transition zones, unreacted materials, pores and microcracks of concrete were analyzed. It can be seen that there are obvious pores and microcracks in the structure. A large number of unreacted FA and SF particles were observed under high-power scanning electron microscopy, indicating that the polymerization reaction between the two was not sufficient. Therefore, increasing the content of FA and SF reduces the compressive strength of the geopolymer structure. However, appropriate FA and SF particles can fill the tiny pores near the interfacial transition zone, improve the pore structure of the concrete, and provide a more stable internal structure.

## 4. Conclusions

In this study, CGS is innovatively utilized as the primary material for preparing geopolymer concrete, with a sodium silicate solution serving as the alkali activator. Simultaneously, a significant amount of powdered waste is employed to replace traditional cementitious materials, such as cement, which are environmentally detrimental during production. This approach aims to achieve the resource utilization of coal-based solid waste, contributing to sustainable construction practices.

(1) This study focuses on CGS as the research subject. Through testing its particle size distribution, chemical composition, and mineral constituents, it was found that CGS exhibits characteristics such as low density, uneven particle distribution, high specific surface area, weak alkalinity, and strong water absorption. Based on determining the material’s Si/Al and Ca/Si ratios, a mixture proportion design was developed using a simplex centroid design method, incorporating CGS, FA, SF, and lime as cementitious materials. The ratios were set within the following ranges: Si/Al (2.8–3.5), and Ca/Si (0.3–0.5). The water-to-binder ratio was set at 0.35 and the binder-to-sand ratio was set at 1.0. Sodium silicate was selected as the alkali activator, and three different moduli (1.0, 1.5, 2.0) were chosen for experimental testing.
(2) Based on experimental results, the mechanical strength of concrete is jointly influenced by the modulus of sodium silicate (sodium silicate) and the relative proportions of raw materials. Additionally, the dry density and curing age of concrete also affect its mechanical properties. For alkali-activated coal gasification slag concrete under different activator moduli, compressive performance is primarily governed by the combined effects of SF content, CGS content, and FA content. When the modulus is 1.0 or 1.5, the flexural strength of the concrete is predominantly influenced by SF content, with SF significantly enhancing flexural strength. In contrast, CGS and FA contribute minimally to improving flexural strength.
(3) Research on compressive, flexural, and tensile strengths of specimens with different sizes revealed that the compressive failure process consists of three distinct stages: the elastic phase, crack propagation phase, and failure phase. Analysis of load-displacement data showed that the stress-strain curves at failure exhibited characteristics similar to conventional concrete. Through fitting compressive strength data from 100 mm and 20 mm cubic specimens, the size conversion coefficient was determined to be approximately 0.456 for compressive strength and 0.599 for flexural strength. After applying a size effect conversion, the 28-day compressive strength of specimens ranged from 7 MPa to 11.97 MPa, while flexural strength varied between 1.95 MPa and 5.58 MPa. Both the Brazilian splitting test and double punch test methods proved effective for indirect measurement of splitting tensile strength. The results obtained from these two methods showed good agreement, demonstrating their reliability in characterizing the tensile properties of alkali-activated CGS concrete.

## Figures and Tables

**Table 1:** Particle size distribution of coal gasification slag from sieve analysis.

**Size of Screen Mesh (mm)**	9.5	4.75	2.36	1.18	0.6	0.3	0.15	0.075
**Percent of Pass (%)**	100	94.09	73.38	55.89	40.65	16.63	6.37	1.27

**Table 2:** Physical properties of coal gasification slag.

Surface Area (m^2^/g)	pH	Burning Loss (%)	Density (g/cm^3^)	Water Absorption (%)
84.50	9.33	3.3	1.63	4.0

**Table 3:** Chemical constituents of cementitious materials used in experiment (Mass%).

Material	SiO_2_	Al_2_O_3_	Fe_2_O_3_	CaO	MgO	Na_2_O	K_2_O
CGS	41.19	15.46	17.70	12.61	1.68	1.71	1.36
FA	42.43	21.38	12.81	1.12	2.12	2.04	1.02
SF	96.78	0.78	0.56	0.64	0.73	0.78	0.67

Coal gasification slag: CGS, fly ash: FA, silica fume: SF.

**Table 4:** Chemical composition and basic properties of sodium silicate solution.

**Chemical Composition (%)**			**Density(g/cm^3^)**	**Modulus**	**pH**
Na_2_O	SiO_2_	H_2_O	1.47	3.30	10–13
7.96	26.10	65.94			

**Table 5:** The relative composition of four cementitious materials and their Si/Al and Ca/Si ratios.

NO	Mass Ratio				Molar Ratio	
	CGS	FA	SF	LF	Si/Al	Ca/Si
a	0.18	0.5	0.2	0.12	3.46	0.32
b	0.3	0.4	0.1	0.2	2.90	0.48
c	0.35	0.3	0.15	0.2	3.45	0.43
d	0.45	0.25	0.1	0.2	3.11	0.48
e	0.5	0.2	0.1	0.2	3.18	0.47

**Table 6:** Mix design proportions (kg/m^3^).

No.	Modulus	CGS	FA	SF	LF	R.S	Water	S.S	W/B
1	1.0	180	500	200	120	1000	125	300	0.35
2	1.0	300	400	100	200	1000	125	300	0.35
3	1.0	350	300	150	200	1000	125	300	0.35
4	1.0	450	250	100	200	1000	125	300	0.35
5	1.0	500	200	100	200	1000	125	300	0.35
6	1.5	180	500	200	120	1000	95	350	0.35
7	1.5	300	400	100	200	1000	95	350	0.35
8	1.5	350	300	150	200	1000	95	350	0.35
9	1.5	450	250	100	200	1000	95	350	0.35
10	1.5	500	200	100	200	1000	95	350	0.35
11	2.0	180	500	200	120	1000	60	400	0.35
12	2.0	300	400	100	200	1000	60	400	0.35
13	2.0	350	300	150	200	1000	60	400	0.35
14	2.0	450	250	100	200	1000	60	400	0.35
15	2.0	500	200	100	200	1000	60	400	0.35

**Table 7:** Mechanical properties (compressive and flexural strength) at 7, 14 and 28 days.

NO.	*f_c_* (MPa)			*f_f_* (MPa)		
Age	7 d	14 d	28 d	7 d	14 d	28 d
1	16.73	22.29	24.08	1.83	3.19	3.28
2	16.86	20.42	23.42	2.90	3.78	5.92
3	17.65	20.41	21.82	2.03	3.52	6.57
4	14.00	15.77	18.99	1.60	3.11	5.76
5	15.21	21.45	22.26	2.13	5.87	7.49
6	12.19	16.15	26.25	1.89	3.39	4.67
7	10.44	15.24	22.69	2.40	3.78	5.33
8	12.50	18.81	23.26	1.86	3.26	6.17
9	6.06	10.06	15.36	1.84	5.04	7.97
10	10.26	17.17	21.39	3.36	6.71	9.31
11	10.79	12.85	16.06	3.44	5.45	6.13
12	11.17	16.89	24.61	3.10	5.49	7.29
13	10.03	17.31	18.27	1.83	3.19	3.28
14	11.90	15.79	21.13	2.90	3.78	5.92
15	12.01	16.42	17.31	2.03	3.52	6.57

**Table 8:** Test results for average unconfined compressive strength of concrete at 28 days.

Mix Scheme	1	2	3	6	7	8	11	12	13
*f*_cu_ (MPa)	14.83	14.14	13.47	8.73	11.80	7.30	5.61	8.39	7.05

**Table 9:** Calculation of shear strength index of CGS concrete.

Mix Scheme	1	2	3	6	7	8	11	12	13
*C*(MPa)	1.42	1.43	1.42	1.38	1.36	1.30	1.24	1.28	1.20
$\varphi {(}^{\circ })$	42.73	41.56	40.67	31.3	38.71	28.34	22.76	32.16	29.47

## Data Availability

The original contributions presented in this study are included in the article. Further inquiries can be directed to the corresponding author(s).
